# Supplementary material for: A small periplasmic protein governs broad physiological adaptations in Vibrio cholerae via regulation of the DbfRS two-component system
Source: Nat Commun. 2025 Dec 18;16:11230. doi: 10.1038/s41467-025-66735-3 (PMC12714742; doi:10.1038/s41467-025-66735-3)
Supplement: Supplementary file 1 — Supplementary Information [file 41467_2025_66735_MOESM1_ESM.pdf]

Supplementary Information for

A small periplasmic protein governs broad physiological adaptations in *Vibrio cholerae* via regulation of the DbfRS two-component system

Emmy Nguyen, Charles Agbavor, Anjali Steenhaut, M. R. Pratyush, N. Luisa Hiller, Laty A. Cahoon, Irina V. Mikheyeva, Wai-Leung Ng & Andrew A. Bridges*

* Corresponding author: bridges@cmu.edu

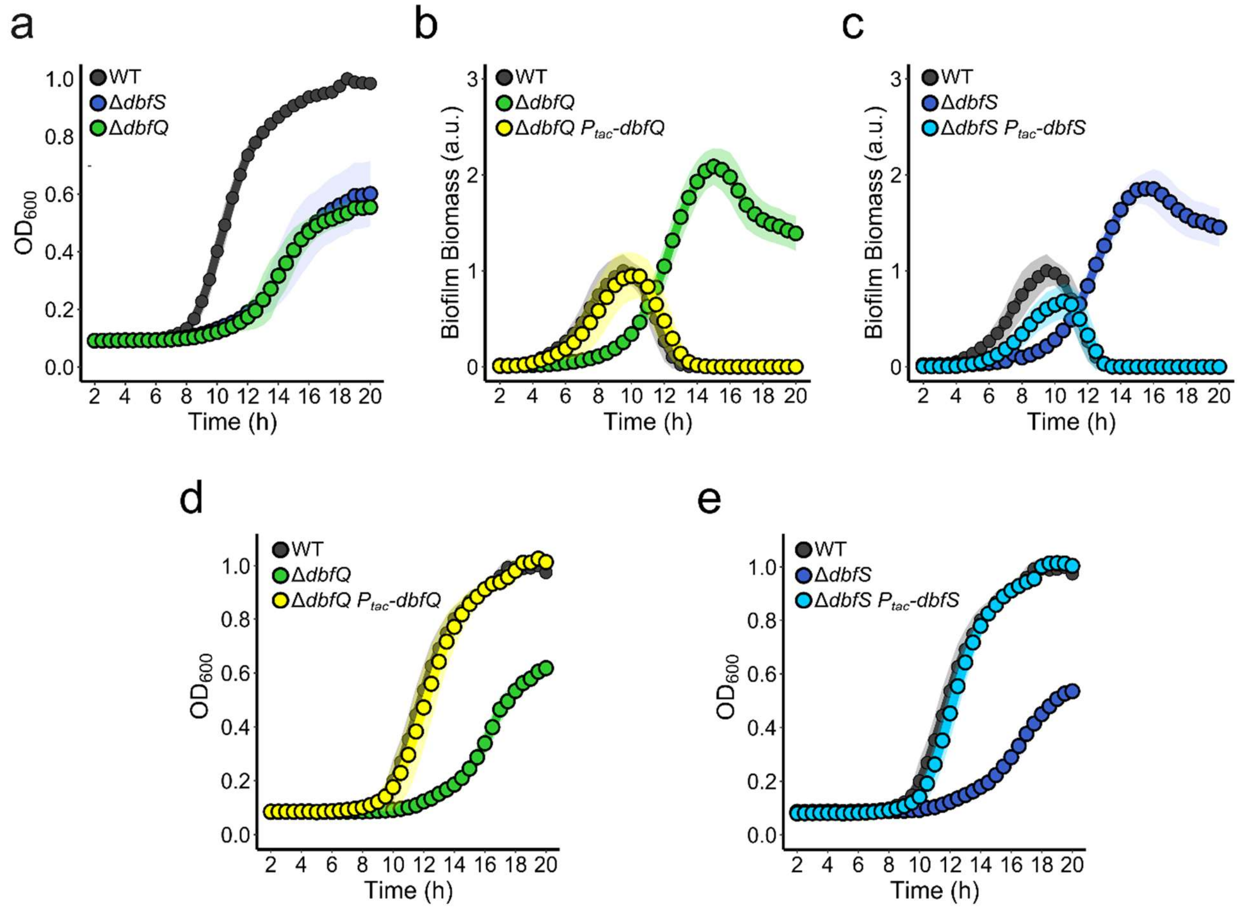

Supplementary Figure 1: Δ*dbfQ* and Δ*dbfS* growth curves and complementation. a Growth curves of WT *V. cholerae* compared to the Δ*dbfQ* and Δ*dbfS* mutant strains as measured by OD_{600}. **b** Quantification of biofilm biomass over time using time-lapse brightfield microscopy for WT, Δ*dbfQ*, and Δ*dbfQ* P_{tac}-*dbfQ* expressed from an ectopic locus (*vc_1807*). **c** As in (**b**) for WT, Δ*dbfS*, and the Δ*dbfS* P_{tac}-*dbfS* complemented strain. **d** As in (**a**) for WT, Δ*dbfQ*, and Δ*dbfQ* P_{tac}-*dbfQ* complemented strain. **e** As in (**a**) for WT, Δ*dbfS*, and Δ*dbfS* P_{tac}-*dbfS* complemented strain. For all panels, points represent averages of *N* = 2 biological replicates and 3 technical replicates, ± SD (shaded regions). a.u. arb. units.

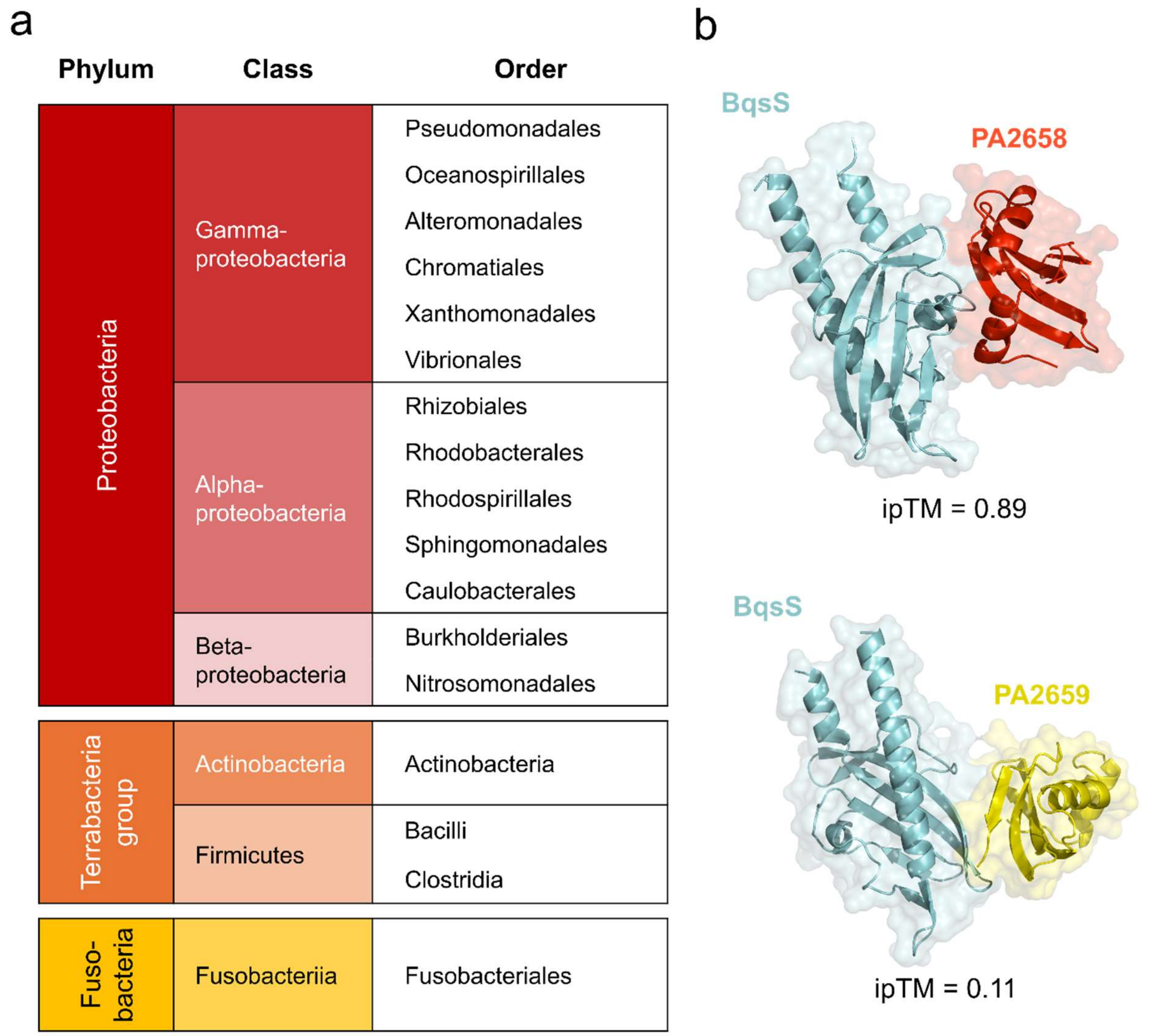

Supplementary Figure 2: **Phylogenomic conservation of DbfQRS-like gene neighborhood and AlphaFold3-predicted interactions from *Pseudomonas aeruginosa.* a** Representative taxa encoding a PepSY domain-containing protein and a response regulator or histidine kinase within a 4-gene vicinity**.** All taxa (phyla, classes, and orders) that have a representation of 0.8% or higher are shown. Phyla are arranged from top to bottom by order of representation. A wide range of bacterial taxa encodes this module, with a large representation of γ-, α- and β-proteobacteria, actinobacteria, firmicutes, and a smaller representation of fusobacteria. **b** AlphaFold3 predictions for the mature (signal peptide–cleaved) PepSY-domain containing proteins PA2658 (beginning at residue 25) and PA2659 (beginning at residue 23) modeled against the sensory domain of the histidine kinase BqsS (PA2656, residue 25-152). Top panel: PA2658 (red)–BqsS (cyan), with an ipTM = 0.89. Bottom panel: PA2659 (yellow) – BqsS (cyan), with an ipTM = 0.11

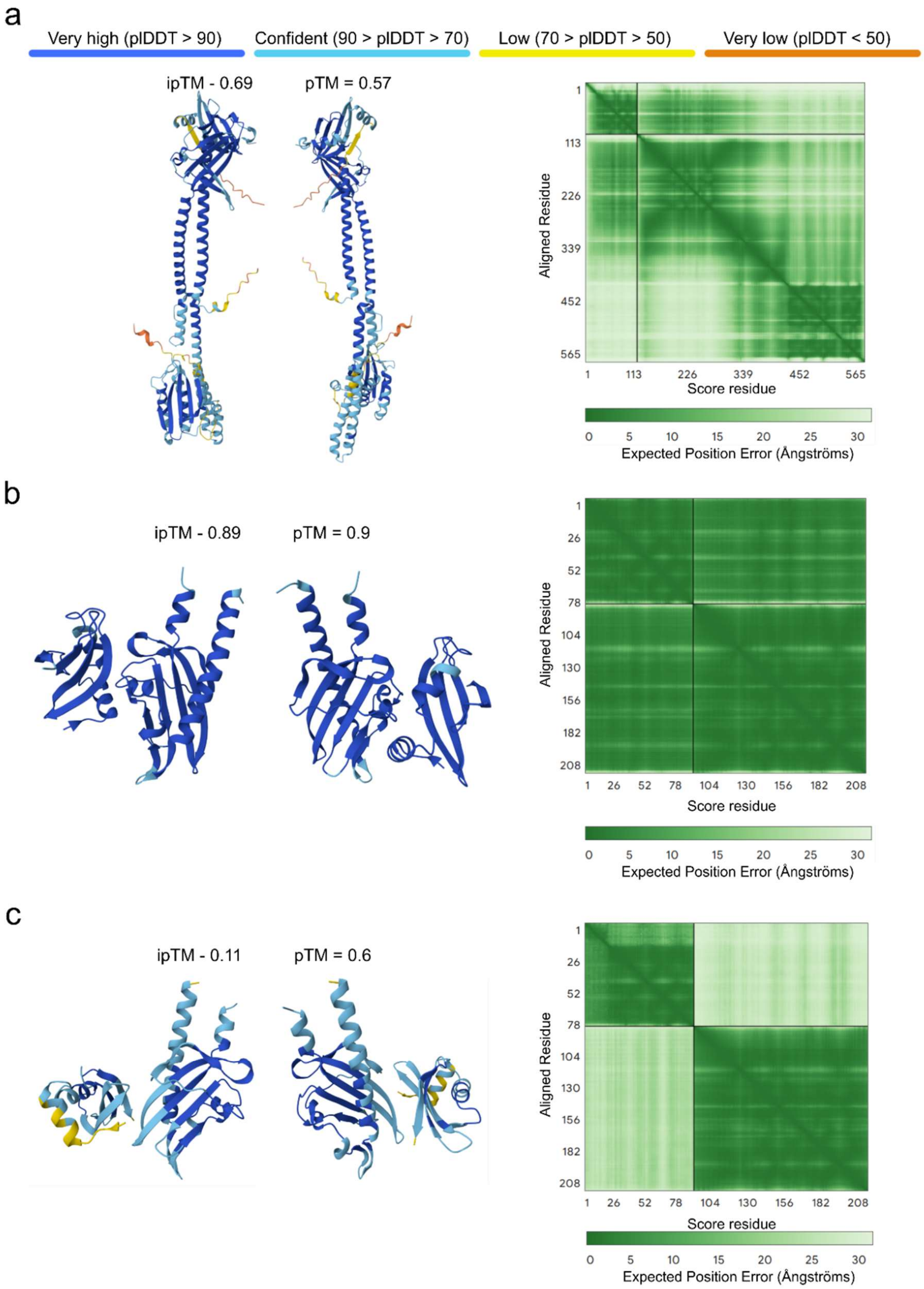
a
Very high (plDDT > 90)
Confident (90 > plDDT > 70)
Low (70 > plDDT > 50)
Very low (plDDT < 50)
ipTM - 0.69
pTM = 0.57
Aligned Residue
1 113 226 339 452 565
Score residue
0 5 10 15 20 25 30
Expected Position Error (Ångströms)
b
ipTM - 0.89
pTM = 0.9
Aligned Residue
1 26 52 78 104 130 156 182 208
Score residue
0 5 10 15 20 25 30
Expected Position Error (Ångströms)
c
ipTM - 0.11
pTM = 0.6
Aligned Residue
1 26 52 78 104 130 156 182 208
Score residue
0 5 10 15 20 25 30
Expected Position Error (Ångströms)

Supplementary Figure 3: AlphaFold3 models of PepSY domain-containing protein(s) (DbfQ-like) and their cognate histidine kinase (DbfS-like). a Predicted DbfQ–DbfS complex from *V. cholerae*, shown with the per-residue measure of local confidence scores (pLDDT) overlaid from two viewing angles and the corresponding Predicted Aligned Error (PAE). **b** As in (**a**) for PA2658-BqsS complex from *P. aeruginosa*. **c** As in (**a**) for PA2659-BqsS complex from *P. aeruginosa*.

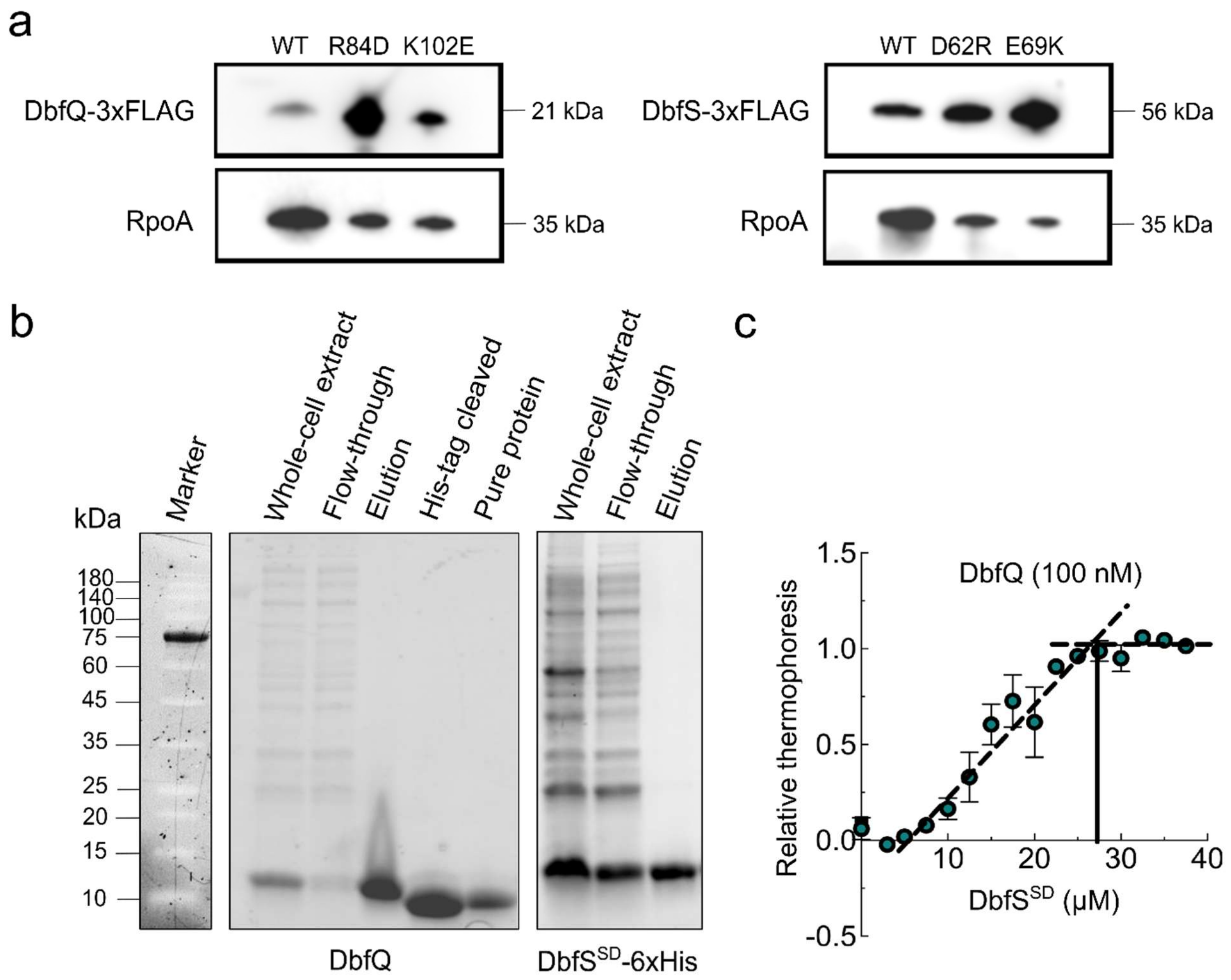

Supplementary Figure 4: DbfQ-DbfS interaction: expression, purification, and binding stoichiometry. a Top panels: Western blot results showing 3xFLAG-tagged DbfQ variants (Left: WT, R84D, K102E) and 3xFLAG-tagged DbfS variants (Right: WT, D62R, E69K). Bottom panels: RpoA serves as the loading control. Because the *dbfQRS* operon is autoregulated and WT expression is low, WT lysates were loaded at 5x the amount of the point-mutant lysates to enable visualization. Data are representative of *N* = 3 biological replicates. **b** Representative SDS-PAGE gel analyses of DbfQ-6×His and DbfS^{SD}-6×His protein purifications. The 6×His tag of DbfQ was removed by thrombin cleavage for pull-down assays. **c** Microscale thermophoresis of fluorescently labeled DbfQ titrated with increasing concentrations of DbfS^{SD}. The dashed lines indicate the saturation point (“kink”) consistent with an apparent binding ratio of 1:1. Data are presented as relative thermophoresis values ± SD of *N* = 4 independent measurements.

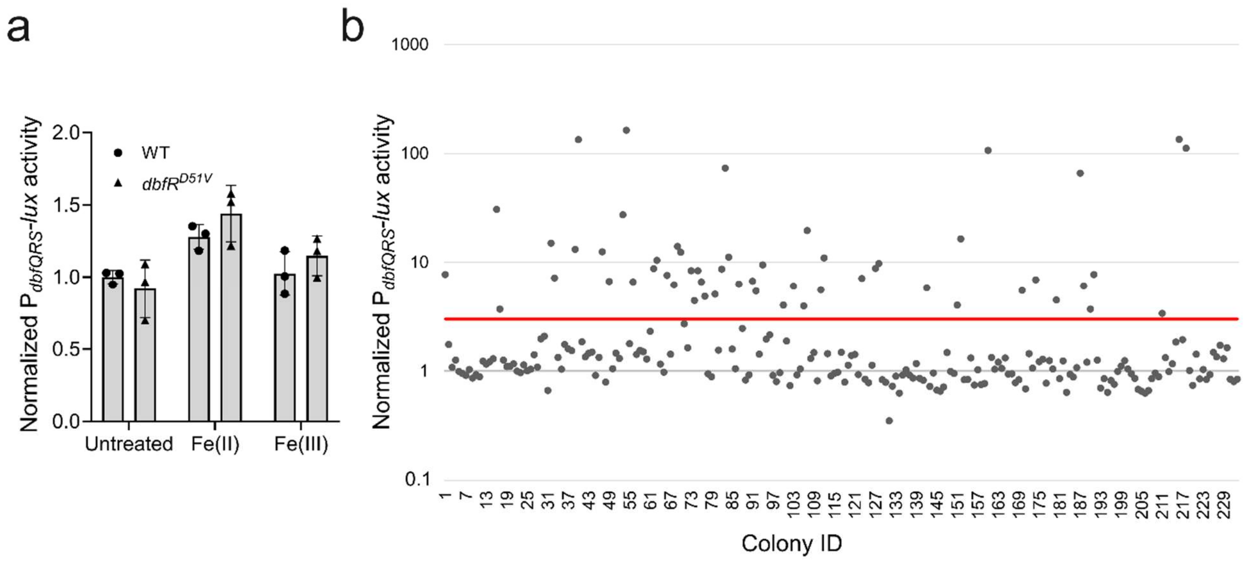

Supplementary Figure 5: Identification of activators of the DbfQRS pathway. a P_{dbfQRS}-*lux* reporter outputs in WT and *dbfR*^{D51V} strains remain unchanged (<3-fold increase in RLU) in the presence of 100 μM Fe(II) or Fe(III). Data are presented as mean ± SD of peak RLU normalized to the average peak value of WT. Points represent individual replicates of *N* = 3 biological replicates. **b** Transposon mutagenesis screen for P_{dbfQRS}-*lux* reporter activation**.** Visual screening of ~20,000 colonies yielded 232 colonies with elevated luminescence compared to the parent strain. Luminescence outputs (relative light units, RLU) for these strains were subsequently quantified using a Biotek Cytation 1 plate reader and normalized to the WT parental strain (normalized RLU = 1). The scatter plot shows the normalized RLU values for all 232 colonies, with a red solid line indicating a 3-fold increase in RLUs. A total of 54 colonies exceeding this threshold were selected for transposon insertion site sequencing.

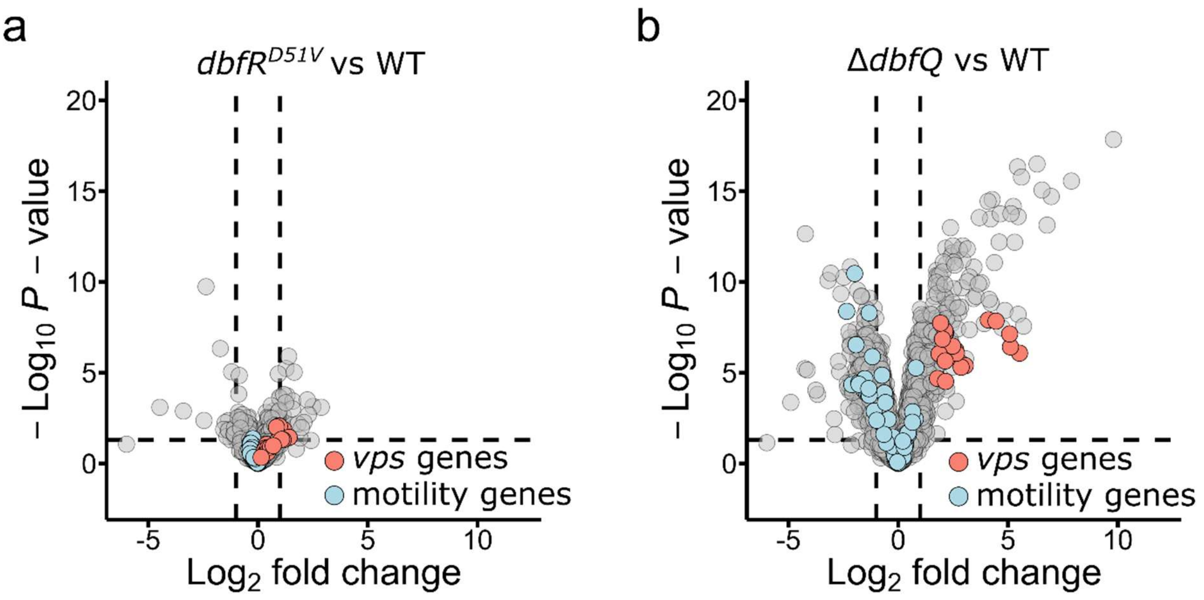

Supplementary Figure 6: RNA sequencing results for *dbfR*^{D51V} and Δ*dbfQ* strains. **a** Volcano plot comparing fold changes and P-values for gene expression in the *dbfR*^{D51V} *V. cholerae* strain compared to WT. *vps* and motility genes are colored in orange and blue, respectively. The horizontal dotted line represents a -Log_{10} *P*-value of 0.05 and left and right vertical dashed lines represent Log_2 fold changes of −1 and 1, respectively. Samples were collected at OD_{600} = 0.1 and *N* = 3 biological replicates for each strain. Complete datasets are available in Supplementary Data 1. **b** As in (**a**) for the Δ*dbfQ* strain compared to WT. Complete datasets are available in Supplementary Data 3.

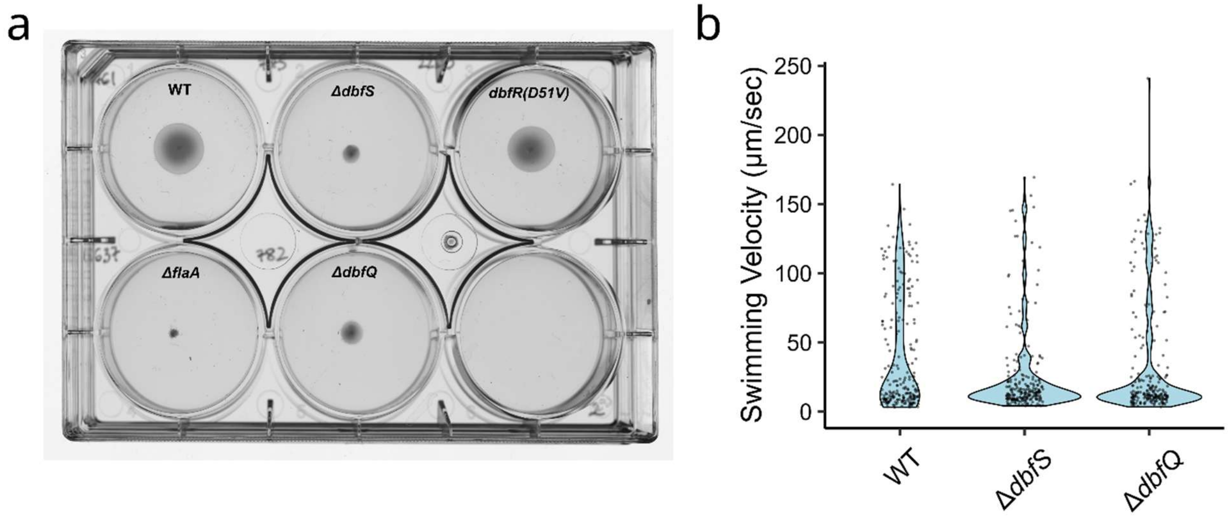

Supplementary Figure 7: ∆*dbfQ* and ∆*dbfS* mutants exhibit decreased motility compared to WT *V. cholerae.* a Images of soft agar plate (LB media, 0.3% agar) motility assays. Representative image was captured after 10 hours of growth at 37°C. Results are representative of those from *N* = 6 biological replicates. **b** Results for single-cell motility assays for the indicated strains. For ∆*dbfS* and ∆*dbfQ* mutant cells, >60% of cells exhibited swimming velocities less than 20 um/sec. Points represent computed median velocities for individual trajectories and violin plots display the distribution of track velocities. *N* = 225 tracks for each group.

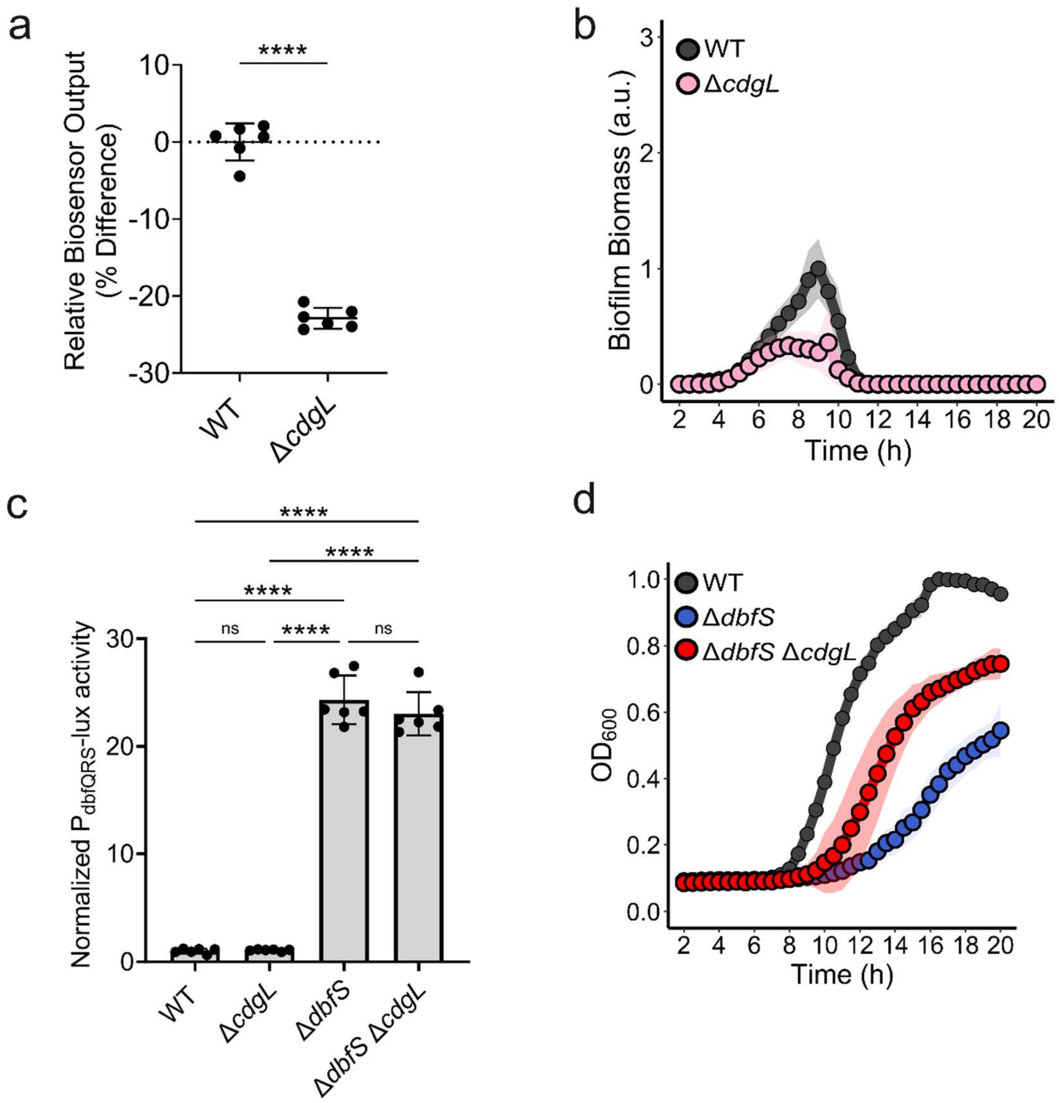

Supplementary Figure 8: CdgL functions downstream of DbfQRS to control intracellular c-di-GMP and biofilm biomass levels. a Relative c-di-GMP reporter output for the WT and Δ*cdgL* strains, expressed as means ± SD of the percentage difference relative to the WT control. Statistical analysis was performed using an unpaired, two-sided t-test with a 95% confidence interval. $P < 0.0001$. **b** Quantification of biofilm biomass for WT and Δ*cdgL* strains using time-lapse brightfield microscopy. **c** Quantification of P_{dbfQRS}-*lux* reporter activity for the indicated strains. Data are presented as mean ± SD of peak RLU normalized to the average peak value of WT. Statistical analysis was performed using one-way ANOVA ($P < 10^{-5}$) with Tukey's multiple comparisons test. From top to bottom, $P < 10^{-5}$, $< 010^{-5}$, $< 10^{-5}$, $= 0.9998$, $< 10^{-5}$ and $= 0.4701$ for the indicated pairs. ****, $P < 0.0001$; ns, not significant. **d** Growth curves of WT *V. cholerae* compared to the Δ*dbfS* and Δ*dbfS* Δ*cdgL* mutant strains as measured by OD_{600}. For all panels, points represent averages of $N = 2$ biological replicates and 3 technical replicates, ± SD.

Supplementary Table 1. List of transposon mutagenized genes resulting in elevated P_{dbfQRS}-*lux* activity.

Gene	Predicted Function	Times hit
**LPS biosynthesis**		
wavA (*vc_0223)*	HepIII transferase	2
wavB (*vc_0224)*	β1,4-glucosyl transferase	1
waaC (*vc_0225)*	HepI transferase	2
wavJ (*vc_0235)*	HepIV transferase	1
waaL (*vc_0237)*	O-antigen ligase	7
wavK (*vc_0238)*	O-acetyltransferase	6
wavL (*vc_0239)*	Glycosyltransferase	11
gmhD (*vc_0240)*	ADP-L-glycero-D-mannoheptose-6-epimerase	3
**Transcription, Translation, and DNA repair**		
vc_r004	16S ribosomal RNA	1
vc_r023	23S ribosomal RNA	2
vc_1130	DNA-binding protein H-NS	1
vc_1392	Deoxyribodipyrimidine photolyase	1
vc_1640	Ribosomal protein L25	1
vc_2030	Ribonuclease E	1
**Transport**		
lysW (*vc_1131)*	Na+/H+ antiporter NhaC family protein	1
epsL (*vc_2725)*	Type II secretion system protein L	1
**Signal transduction**		
dbfQ (*vc_1637)*	PepSY domain-containing protein	2
dbfS (*vc_1639)*	Histidine kinase	4
**Metabolism**		
vc_0550	Oxaloacetate decarboxylase	1
**Hypothetical proteins**		
vc_a0092		1
vc_0180		1

**Phage shock protein**		
pspA (*vc_1678*)	Phage shock protein A	2
**Virulence**		
rtxA (*vc_1451)*	RTX toxin RtxA	1
**Total**		**54**

Supplementary Table 2: Gene disruptions identified as suppressing Δ*dbfS* colony rugosity.

Gene	Common Name	Hit Frequency
vc_0917	*vpsA*	14
vc_0916	*vpsU*	12
vc_0922	*vpsF*	12
vc_0665	*vpsR*	10
vc_0935	*vpsM*	10
vc_2285	*cdgL*	10
vc_0921	*vpsE*	9
vc_0925	*vpsI*	9
vc_0923	*vpsG*	8
vc_0924	*vpsH*	8
vc_0937	*vpsO*	8
vc_0934	*vpsL*	7
vc_2529	*rpoN*	7
vc_0920	*vpsD*	6
vc_0926	*vpsJ*	6
vc_0927	*vpsK*	6
vc_a0952	*vpsT*	6
vc_0395	*galU*	4
vc_0918	*vpsB*	4
vc_0919	*vpsC*	4
vc_0237	-	3
vc_0239	-	3
vc_a0774	*galE*	3
vc_1021	*luxO*	2
vc_0601	-	1
vc_0628	-	1
vc_0633	*ompU*	1
vc_0664	*lysS*	1
vc_0928	*rbmA*	1
vc_0932	*rbmE*	1
vc_0936	*vpsN*	1
vc_1609	-	1
vc_2423	*pilA*	1
vc_a0824	*ectB*	1
vc_0901	-	1
