# Supplementary material for: A small periplasmic protein governs broad physiological adaptations in Vibrio cholerae via regulation of the DbfRS two-component system
Source: Nat Commun. 2025 Dec 18;16:11230. doi: 10.1038/s41467-025-66735-3 (PMC12714742; doi:10.1038/s41467-025-66735-3)
Supplement: Supplementary file 2 — Reporting Summary [file 41467_2025_66735_MOESM2_ESM.pdf]

nature portfolio

Corresponding author(s): Andrew A. Bridges

Last updated by author(s): Oct 20, 2025

Reporting Summary

Nature Portfolio wishes to improve the reproducibility of the work that we publish. This form provides structure for consistency and transparency in reporting. For further information on Nature Portfolio policies, see our Editorial Policies and the Editorial Policy Checklist.

Statistics

For all statistical analyses, confirm that the following items are present in the figure legend, table legend, main text, or Methods section.

n/a	Confirmed	
☐	☒	The exact sample size (*n*) for each experimental group/condition, given as a discrete number and unit of measurement
☐	☒	A statement on whether measurements were taken from distinct samples or whether the same sample was measured repeatedly
☐	☒	The statistical test(s) used AND whether they are one- or two-sided<br>*Only common tests should be described solely by name; describe more complex techniques in the Methods section.*
☒	☐	A description of all covariates tested
☒	☐	A description of any assumptions or corrections, such as tests of normality and adjustment for multiple comparisons
☐	☒	A full description of the statistical parameters including central tendency (e.g. means) or other basic estimates (e.g. regression coefficient) AND variation (e.g. standard deviation) or associated estimates of uncertainty (e.g. confidence intervals)
☐	☒	For null hypothesis testing, the test statistic (e.g. *F*, *t*, *r*) with confidence intervals, effect sizes, degrees of freedom and *P* value noted<br>*Give P values as exact values whenever suitable.*
☒	☐	For Bayesian analysis, information on the choice of priors and Markov chain Monte Carlo settings
☒	☐	For hierarchical and complex designs, identification of the appropriate level for tests and full reporting of outcomes
☐	☒	Estimates of effect sizes (e.g. Cohen's *d*, Pearson's *r*), indicating how they were calculated

Our web collection on statistics for biologists contains articles on many of the points above.

Software and code

Policy information about availability of computer code

Data collection	Commercial software was used for data collection, namely Biotek Gen5 (version 3.12) software and Leica LasX software (version 3.7).
Data analysis	Data analyses were performed using the same data collection softwares described above, GraphPad Prism (version 10.4.1), PyMol (version 3.1), FIJI (version 1.54k), RStudio (version 4.4.1), along with previously published image analysis pipelines in the Julia programming language (version 1.11.1). Figures and original cartoons were assembled in Inkscape software (version 1.4). Analysis and softwares used are described in the methods of the manuscript.

For manuscripts utilizing custom algorithms or software that are central to the research but not yet described in published literature, software must be made available to editors and reviewers. We strongly encourage code deposition in a community repository (e.g. GitHub). See the Nature Portfolio guidelines for submitting code & software for further information.

Data

Policy information about availability of data

All manuscripts must include a data availability statement. This statement should provide the following information, where applicable:

- Accession codes, unique identifiers, or web links for publicly available datasets
- A description of any restrictions on data availability
- For clinical datasets or third party data, please ensure that the statement adheres to our policy

The source data used to generate all main and supporting figures in this work are available on Figshare (https://doi.org/10.1184/R1/28653392), or as in Supplementary Data files.

Research involving human participants, their data, or biological material

Policy information about studies with human participants or human data. See also policy information about sex, gender (identity/presentation), and sexual orientation and race, ethnicity and racism.

Reporting on sex and gender	N/A
Reporting on race, ethnicity, or other socially relevant groupings	N/A
Population characteristics	N/A
Recruitment	N/A
Ethics oversight	N/A

Note that full information on the approval of the study protocol must also be provided in the manuscript.

Field-specific reporting

Please select the one below that is the best fit for your research. If you are not sure, read the appropriate sections before making your selection.

[x] Life sciences [ ] Behavioural & social sciences [ ] Ecological, evolutionary & environmental sciences

For a reference copy of the document with all sections, see nature.com/documents/nr-reporting-summary-flat.pdf

Life sciences study design

All studies must disclose on these points even when the disclosure is negative.

Sample size	No formal power calculations were performed to predetermine sample size. Instead, sample sizes were selected in accordance with established practices in microbiology and prior work in Vibrio cholerae pathogenesis models. For in vitro assays, a minimum of three independent biological replicates were performed, while for in vivo colonization assays at least five infant mice were used per condition, with each mouse considered an individual biological replicate. These sample sizes were considered sufficient to capture reproducible biological effects while adhering to ethical guidelines for animal use.
Data exclusions	No data were excluded in this study.
Replication	All data presented in the manuscript are derived from both biological and technical replicates that were performed independently to ensure reproducibility and robustness of the findings. Biological replicates were conducted using separate cultures or individual animals, while technical replicates involved repeated measurements from the same sample under identical experimental conditions.
Randomization	Not applicable, as this study does not involve analysis that requires randomization.
Blinding	Blinding was not relevant in this study, computational analysis was performed in an unbiased manner across all test conditions.

Reporting for specific materials, systems and methods

We require information from authors about some types of materials, experimental systems and methods used in many studies. Here, indicate whether each material, system or method listed is relevant to your study. If you are not sure if a list item applies to your research, read the appropriate section before selecting a response.

Materials & experimental systems

n/a	Involved in the study	
[ ]	[x]	Antibodies
[x]	[ ]	Eukaryotic cell lines
[x]	[ ]	Palaeontology and archaeology
[ ]	[x]	Animals and other organisms
[x]	[ ]	Clinical data
[x]	[ ]	Dual use research of concern
[x]	[ ]	Plants

Methods

n/a	Involved in the study	
[x]	[ ]	ChIP-seq
[x]	[ ]	Flow cytometry
[x]	[ ]	MRI-based neuroimaging

Antibodies

Antibodies used	1. Anti-FLAG-Peroxidase antibody (Millipore Sigma, #A8592), used for Western Blotting at 1:5000 dilution;<br>2. Anti-Escherichia coli RNA Polymerase α (Biolegend, #663104), used for Western Blotting at 1:10,000 dilution;

	3. Anti-Mouse IgG HRP conjugated antibody (Promega, #W4021), used for Western Blotting at 1:10,000 dilution.
Validation	These antibodies have been extensively used in past studies. The specificity of the anti-FLAG antibody was verified to be non-reactive again strains that do not encode the flag epitope. The specificity of Anti-Mouse IgG HRP conjugated antibody was verified to be non-reactive in the absence of IgG.

Animals and other research organisms

Policy information about studies involving animals; ARRIVE guidelines recommended for reporting animal research, and Sex and Gender in Research

Laboratory animals	Infant CD-1 mice (Charles River Laboratories), 4–7 days old, were used for in vivo competition assays.
Wild animals	This study did not involve wild animals.
Reporting on sex	The sex of neonatal CD-1 mice was not determined at the time of use. Both sexes were randomly included in experiments. Sex-based analyses were not performed as there are no known sex-dependent differences in this infant mouse colonization model.
Field-collected samples	This study did not involve samples collected from the field.
Ethics oversight	All animal experiments were approved by the Institutional Animal Care and Use Committee (IACUC) at Tufts University School of Medicine (Protocol B2024-26). Animals were housed in an AAALAC-accredited facility under the oversight of Comparative Medicine Services at Tufts University School of Medicine.

Note that full information on the approval of the study protocol must also be provided in the manuscript.

Plants

Seed stocks	*Report on the source of all seed stocks or other plant material used. If applicable, state the seed stock centre and catalogue number. If plant specimens were collected from the field, describe the collection location, date and sampling procedures.*
Novel plant genotypes	*Describe the methods by which all novel plant genotypes were produced. This includes those generated by transgenic approaches, gene editing, chemical/radiation-based mutagenesis and hybridization. For transgenic lines, describe the transformation method, the number of independent lines analyzed and the generation upon which experiments were performed. For gene-edited lines, describe the editor used, the endogenous sequence targeted for editing, the targeting guide RNA sequence (if applicable) and how the editor was applied.*
Authentication	*Describe any authentication procedures for each seed stock used or novel genotype generated. Describe any experiments used to assess the effect of a mutation and, where applicable, how potential secondary effects (e.g. second site T-DNA insertions, mosiacism, off-target gene editing) were examined.*
