# Supplementary material for: A small periplasmic protein governs broad physiological adaptations in Vibrio cholerae via regulation of the DbfRS two-component system
Source: Nat Commun. 2025 Dec 18;16:11230. doi: 10.1038/s41467-025-66735-3 (PMC12714742; doi:10.1038/s41467-025-66735-3)
Supplement: Supplementary file 3 — Description of Additional Supplementary Files [file 41467_2025_66735_MOESM3_ESM.pdf]

Description of Additional Supplementary Files:

Supplementary Data 1: RNAsequencing results comparing *dbfR*^{D51V} and WT *V. cholerae* strains.

Supplementary Data 2: RNAsequencing results comparing Δ*dbfS* and WT *V. cholerae* strains.

Supplementary Data 3: RNAsequencing results comparing Δ*dbfQ* and WT *V. cholerae* strains.

Supplementary Data 4: KEGG enrichment analysis results for Δ*dbfS* strain.

Supplementary Data 5: Strains used in this study

Supplementary Data 6: DNA oligonucleotides and gene fragments used in this study
